# Wireless Magnetic-Based Closed-Loop Control of Self-Propelled Microjets

**DOI:** 10.1371/journal.pone.0083053

**Published:** 2014-02-05

**Authors:** Islam S. M. Khalil, Veronika Magdanz, Samuel Sanchez, Oliver G. Schmidt, Sarthak Misra

**Affiliations:** 1 The German University in Cairo, Cairo, Egypt; 2 Institute for Integrative Nanosciences, IFW Dresden, Dresden, Germany; 3 Max Planck Institute for Intelligent Systems, Stuttgart, Germany; 4 University of Technology Chemnitz, Chemnitz, Germany; 5 University of Twente, Enschede, The Netherlands; Tel Aviv University, Israel

## Abstract

In this study, we demonstrate closed-loop motion control of self-propelled microjets under the influence of external magnetic fields. We control the orientation of the microjets using external magnetic torque, whereas the linear motion towards a reference position is accomplished by the thrust and pulling magnetic forces generated by the ejecting oxygen bubbles and field gradients, respectively. The magnetic dipole moment of the microjets is characterized using the *U*-turn technique, and its average is calculated to be 1.3
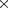
10^−10^ A.m^2^ at magnetic field and linear velocity of 2 mT and 100 µm/s, respectively. The characterized magnetic dipole moment is used in the realization of the magnetic force-current map of the microjets. This map in turn is used for the design of a closed-loop control system that does not depend on the exact dynamical model of the microjets and the accurate knowledge of the parameters of the magnetic system. The motion control characteristics in the transient- and steady-states depend on the concentration of the surrounding fluid (hydrogen peroxide solution) and the strength of the applied magnetic field. Our control system allows us to position microjets at an average velocity of 115 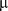
m/s, and within an average region-of-convergence of 365 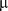
m.

## Introduction

Several types of catalytic [1]–[5] and magnetic [6], [7] microrobots have been demonstrated to overcome Brownian motion at low-Reynolds number regimes [8], [9]. Motion of these microrobots is based on several propulsion mechanisms. Some of these mechanisms depend on the efficient transformation of chemical energy into kinetic energy using catalytic reaction. Gibbs *et al.* [10] presented a model to explain the driving force for spherical colloids, and further showed that the propelling force depends on the surface tension of the liquid and the bulk concentration of hydrogen peroxide. Solovev *et al.* [11] and Mei *et al.* [12] have put forward a propulsion mechanism based on the catalytic decomposition of hydrogen peroxide by microtubular layers of platinum and silver, respectively. It has been also shown that self-propelled microjets can selectively transport relatively large amounts of particles on-a-chip and Murine Cath.a-differentiated cells by controlling the magnetic fields in open-loop [13]. Some propulsion mechanisms are based on pulling the magnetic microrobots with the magnetic field gradients [14]–[18]. However, these mechanisms are limited by the projection distance of the magnetic field gradients. Another propulsion mechanism was proposed by Bell *et al.* [19] and Sendoh *et al.* [20]. This mechanism mimics the morphology of the bacterial flagellum and capitalizes on the generation of rotating magnetic fields. The rotating fields allow the helical microrobot to rotate and act like a corkscrew [8], and hence moves without pulling by the magnetic field gradients.

Biological microrobots [21] have been used by Martel *et al.* to execute non-trivial tasks such as micro-manipulation [22], micro-assembly [23], and micro-actuation [24]. The propulsion mechanism of the biological microrobots depends on rotating their helical flagella to move forward or backward along the external magnetic field lines [25]. This propulsion mechanism allows biological microrobots to benefit from the larger projection distance of the magnetic field. However, the propulsion force of these microrobots is relatively small (10^−12^ N) [26], and this decreases their range of applications. Wang *et al.* [27] has studied the use of both fuel-driven and fuel-free, as well as ultrasound-driven propulsion mechanisms at nano- and micro-scales. Although the previously mentioned propulsion mechanisms provide solutions to key challenges in microrobotic applications, progress towards practical implementation has not yet been accomplished. Substantial efforts have been dedicated to develop and characterize new propulsion mechanisms, whereas only a few attempts have been reported to realize practical applications based on closed-loop (feedback) control systems [28].

**Figure 1:**
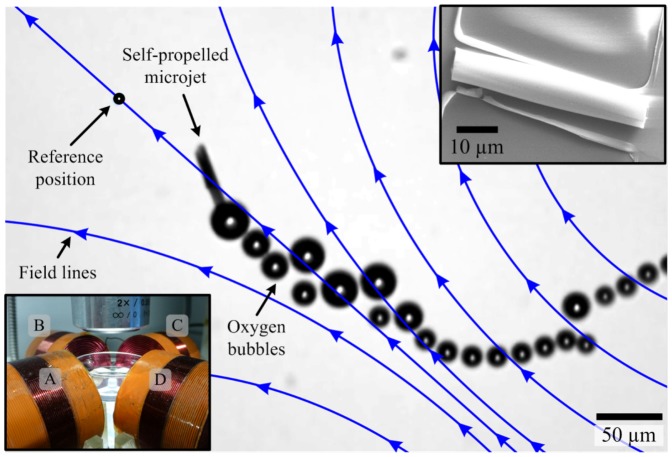
Closed-loop motion control of a self-propelled microjet under the influence of the controlled magnetic fields. Magnetic fields are generated using the magnetic-based manipulation system, shown in the inset at the bottom left corner [14]. The magnetic fields are used to orient the microjet towards the reference position (black circle). The microjet moves towards the reference position using the thrust force generated by the ejecting oxygen bubbles from its end. The velocity of the microjet in this representative experiment is 100 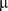
m/s for hydrogen peroxide solution with concentration of 5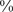. The inset at the upper right corner shows a Scanning Electron Microscopy image of a microjet fixed to its substrate.

In this work, we demonstrate for the first time the closed-loop motion control of self-propelled microjets under the influence of controlled magnetic fields generated by a magnetic-based manipulation system. Figure 1 shows a control exerted over a microjet moving towards a reference position under the influence of the self-propulsion force and the applied magnetic fields. The ejecting oxygen bubbles provide thrust force which allows for the navigation of the microjet along the external magnetic field lines (blue lines). We devise a sliding-mode control system [29] owing to its robustness in the presence of parameter uncertainties and unmodeled disturbance forces such as wall and surface effects, bubbles-microjet interactions, and microjet-microjet interactions. First, the magnetic dipole moment is characterized based on the motion analysis of the microjets using uniform magnetic field reversals [30], [31]. Second, the characterized magnetic dipole moment is used in the realization of a magnetic force-current map of the microjet. The magnetic-based control system capitalizes on this map. Characteristics of our control system are evaluated in the transient- and steady-states using the average velocity and average region-of-convergence of the controlled microjets, respectively. In addition, the control characteristics of the sliding-mode control are compared to the control characteristics of a proportional-derivative (PD) control system. The novelty of our work lies in demonstrating precise point-to-point closed-loop control of self-propelled microjets using weak magnetic fields (2 mT).

## Closed-Loop Motion Control of Self-Propelled Microjets

**Figure 2:**
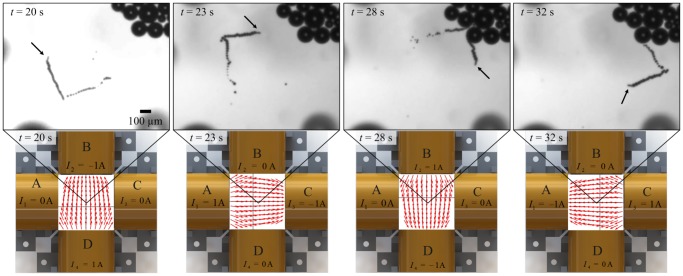
Open-loop motion control of a self-propelled microjet using uniform magnetic fields. The microjet follows a square trajectory with an edge length of 500 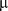
m, at a linear velocity of 100 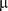
m/s. Uniform magnetic fields are generated using 2 active electromagnets at a time. The bottom row provides finite element (FE) simulations of the uniform magnetic fields utilized in this open-loop control experiment. 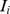 for 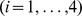, denotes the current at each of the electromagnets. The magnetic field strength within the center of the workspace is 2 mT. The black arrows point at the microjet.

Self-propelled microjets are fabricated by rolled-up nanotech [4], [32] using layers of platinum, titanium, and iron. These microjets are immersed in a solution of hydrogen peroxide (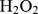) and surrounded by an array of independent electromagnetic coils (Figure 1). The inner platinum layer allows for the catalytic break-down of the hydrogen peroxide into oxygen and water. A thrust force is generated upon the accumulation and release of the oxygen bubbles from one end of the microjet [5]. This force allows the microjet to overcome the Brownian diffusion and the viscous drag forces. The iron layers allow the microjet to orient itself along the external magnetic field lines using magnetic torque. Figure 2 demonstrates the motion of a microjet under the influence of controlled magnetic fields in open-loop. Throughout the experimental work of this study, hydrogen peroxide solution with concentration of 5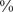 was used, along with small amounts of isopropanol and Triton X. A microjet with a length of 50 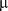
m is immersed in the hydrogen peroxide. Uniform magnetic fields (field strength of 2 mT at the center of the electromagnetic array) are generated using two active electromagnetic coils at a time, and used to achieve a square trajectory of the microjet, as shown in Figure 2. Magnetic fields are measured using a calibrated three-axis Hall magnetometer (Sentron AG, Digital TeslaMeter 3MS1-A2D3-2-2T, Switzerland).

### Characterization and Control Law

**Figure 3:**
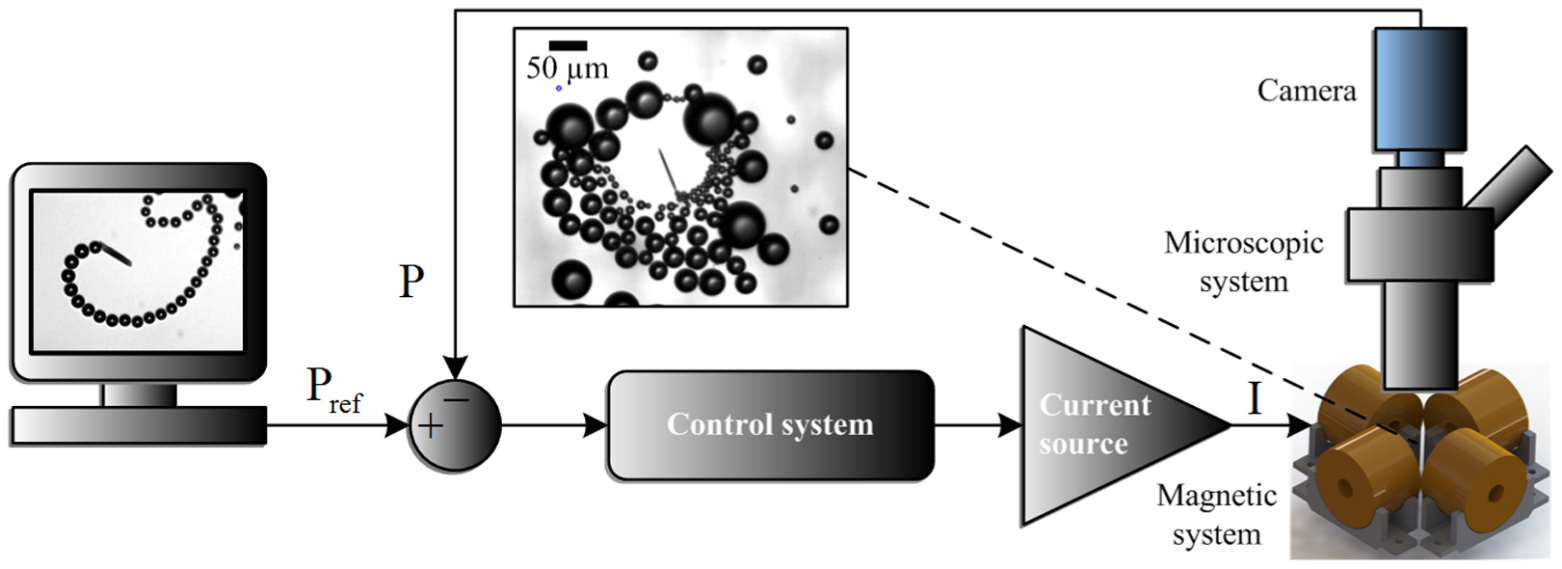
Closed-loop control system for precise positioning of self-propelled microjets (inset). 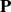
 and 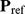 denote the position of the microjet and the reference position, respectively, and 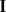 denotes the current vector.

**Figure 4:**
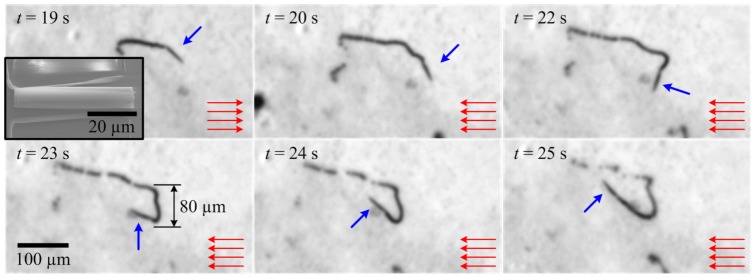
Characterization of the magnetic dipole moment of the self-propelled microjet (blue arrow) using the *U*-turn technique. The microjet follows a *U*-turn trajectory when the magnetic field is reversed. Diameter of the *U*-turn trajectory is used to determine the magnetic dipole moment. In this representative experiment the diameter is 80 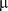
m, and the magnetic dipole moment is calculated to be 1.48
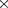
10^−10^A.m^2^ at magnetic field and linear velocity of 2 mT and 100 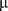
m/s, respectively. The average magnetic dipole moment is 1.31
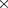
10^−10^A.m^2^, which is deduced from 10 different *U*-turn trials using (3) and used in the realization of the magnetic force-current map (2). The red lines represent the magnetic field lines. The inset shows a Scanning Electron Microscopy image of a microjet fixed to its substrate.

In order to position the self-propelled microjet within the vicinity of a reference point, a closed-loop motion control system is designed (Figure 3). The nominal equation of motion of the microjet is given by
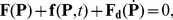
(1)where 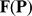, 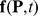, and 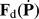 are the magnetic force, self-propulsion force, and drag force of the microjet at point 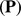, respectively. Our motion control strategy is based on controlling the magnetic force 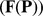 towards a reference position. This control allows the field lines to be directed towards the reference position. A microjet aligns itself along the field lines and moves towards the reference position using its self-propulsion force. The magnetic force is generated using an external magnetic field 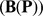, and is given by [16], [26]

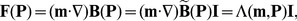
(2)where 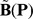 is a matrix that maps the input current 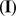 onto magnetic field 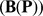. Further, 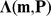 maps the input current onto magnetic force. This force-current map depends on the magnetic dipole moment 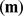 of the microjet and its position 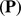. We estimate the magnetic dipole moment of the microjets to realize the magnetic force-current map (2). A microjet aligns itself along the magnetic field lines. Reversing the direction of the magnetic fields causes the microjet to follow a *U*-turn trajectory, as shown in Figure 4. The diameter 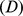 of the *U*-turn trajectory is given by [30], [31]

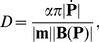
(3)where *α* is the rotational drag coefficient of the microjet, and 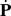 is its linear velocity. In order to determine the magnetic dipole moment of the microjet using (3), uniform magnetic fields are applied using electromagnets A and C (Figure 1). These fields are reversed to initiate *U*-turns of the microjets. We repeated this experiment 10 times and the average *U*-turn diameter is determined from the motion analysis of the microjets. The average magnetic dipole moment is calculated to be 1.31
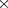
10^−10^A.m^2^, at magnetic field and linear velocity of 2 mT and 100 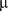
m/s, respectively. In (3), 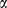 is calculated using [33]

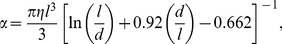
(4)where 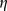 is the dynamic viscosity of the hydrogen peroxide solution. Further, 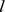 and 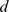 are the length (50 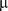
m) and diameter (5 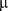
m) of the microjet, respectively.

The magnetic dipole moment is used in the realization of the magnetic force current map (2), this map is a basis of magnetic-based closed-loop control systems. First, we devise the following sliding-mode control input [29]:
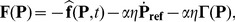
(5)where 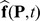 is an estimate of the propulsion force given in (1). Further, 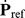 is the reference velocity, and 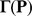 is an additional force input and is given by
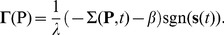
(6)In (6), 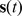 is the sliding-line and is given by
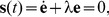
(7)where 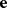 and 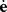 are the position and velocity tracking errors, respectively. Further, 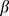 and 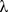 are positive gains. Finally, 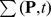 is given by
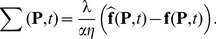
(8)


### Motion Control Results

**Figure 5:**
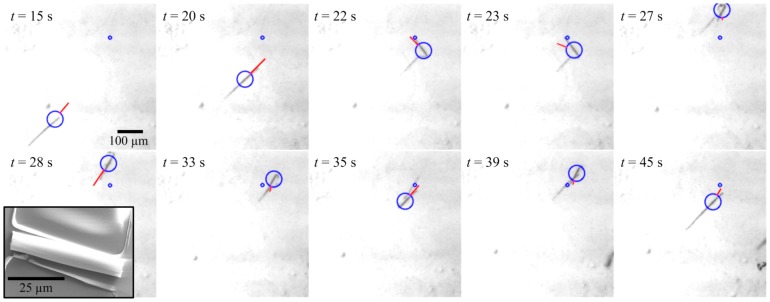
Closed-loop control of a self-propelled microjet using the sliding-mode control law (5). The microjet moves towards a reference position (small blue circle) under the influence of the self-propulsion force and the magnetic fields. The generated magnetic fields using our sliding-mode control system orients the microjet along the magnetic field lines. The microjet moves along these lines using its propulsion force. In this representative experiment, the control system positions the microjet at an average velocity of 90 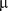
m/s, and within a region-of-convergence of 260 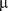
m in diameter. The controller gains are: 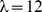 and 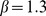. The large blue circle is assigned by our feature tracking software [14] and the red line represents the velocity vector of the microjet. The inset shows a Scanning Electron Microscopy image of a microjet fixed to its substrate.

**Figure 6:**
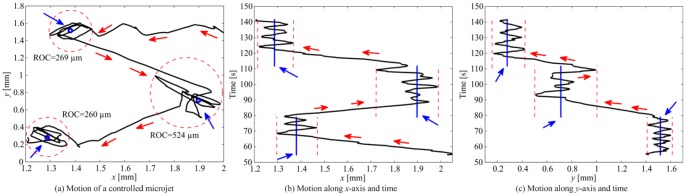
Representative closed-loop control of a self-propelled microjet under the influence of the controlled magnetic fields, using the sliding-mode control law (5). (a) The sliding-mode control system positions the microjet at an average velocity of 97 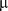
m/s, and within a maximum region-of-convergence (ROC) of 524 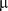
m in diameter. ROCs are represented using the red-dashed circles. The controller gains are: 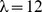 and 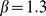. The red arrows indicate the direction of the microjet, whereas the blue arrows indicate the reference positions. (b) Motion of the microjet towards three reference positions (blue vertical lines) along 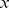-axis. (c) Motion of the microjet along 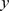-axis.

The sliding-mode control law (5) is implemented by setting (5) equal to (2), and solving for the current vector 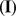 using the pseudoinverse of 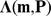. Figure 5 shows a representative closed-loop motion control result of the control law (5). The position error is determined by tracking the motion of the microjet (large blue circle in Figure 5). This position tracking error along with its time derivative are used to calculate 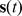. Further, the self-propulsion force estimate 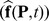 is modeled using a periodic force function with a period of 0.08 second. This period is determined from the motion analysis of the microjets. This time is based on the average elapsed-time of the ejecting oxygen bubbles. Control law (5) allows the magnetic field to orient towards the reference positions (small blue circle). The microjet moves towards the reference position at an average velocity of 90 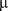
m/s. Due to the self-propulsion force of the microjet, our control system cannot achieve zero position tracking error. It rather positions the microjet within the vicinity of the reference position, which we refer to as a region-of-convergence as shown in Figure 6.

Figure 6 shows the controlled motion of a microjet towards three reference positions. Within the vicinity of the reference, the control system reverses the direction of the magnetic field based on the position and velocity tracking errors (7). This magnetic field reversal allows the microjet to stay within the vicinity of the reference position. Diameter of this region-of-convergence depends on the linear velocity of the microjet, the magnetic torque exerted on its magnetic layers, and the bandwidth of the control system (our microscopic vision system has a maximum frame-per-second of 15). The sliding-mode control gains (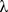 and 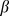) must be positive. The gain 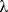 controls the slope of the sliding-line (7), whereas the gain 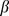 controls the convergence time of the errors (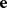 and 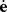) to the sliding-line. In this representative experiment (Figure 6), we observe that setting the controller gains to 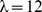 and 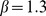, results in an average velocity of 97 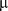
m/s and maximum region-of-convergence of 524 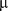
m. This motion control trail is repeated 10 times, and the average velocity and average region-of-convergence are calculated to be 115
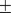
26 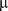
m/s and 356
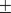
110 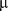
m, respectively.

**Figure 7:**
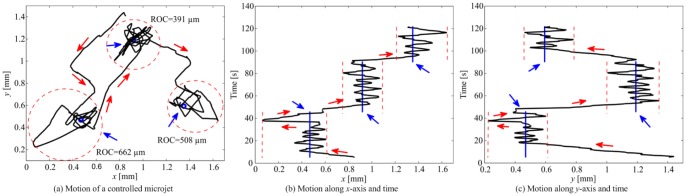
Representative closed-loop control of a self-propelled microjet under the influence of the controlled magnetic fields, using the proportional-derivative (PD) control law (9). (a) The PD control system positions the microjet at an average velocity of 127 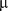
m/s, and within a maximum region-of-convergence (ROC) of 662 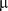
m in diameter. ROCs are represented using the red-dased circles. The controller gains are: 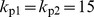 and 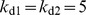, where 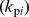 and 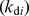 are the entries of the gain matrices 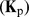 and 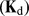 for 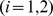, respectively. The red arrows indicate the direction of the microjet, whereas the blue arrows indicate the reference positions. (b) Motion of the microjet towards three reference positions (blue vertical lines) along 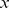-axis. (c) Motion of the microjet along 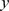-axis.

In order to evaluate the characteristics of the sliding-mode control system, we compare its results to a conventional PD control system. In this case, the magnetic force is given by
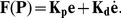
(9)where 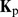 and 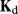 are the controller positive-definite gain matrices. Control law (9) is implemented by setting the PD control force equal to the magnetic force-current map (2), and calculating the current vector using the pseudoinverse of 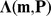 that is based on the characterized magnetic dipole moment and position of the microjet. We observe that the PD control system achieves an average velocity and region-of-convergence of 119
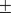
30 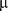
m/s and 417
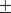
115 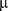
m, respectively. The averages are calculated from 10 closed-loop motion control trials. Figure 7 shows a representative motion control result of the microjet using the PD control system. These results show that the non-linear sliding-mode control system achieves 14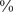 higher positioning accuracy, as opposed to the linear PD control system, in the steady-state.

## Conclusions

Closed-loop control of self-propelled microjets is implemented using a magnetic-based manipulation system and a sliding-mode controller. Magnetic fields are generated and oriented based on the position tracking error of the microjet with respect to the reference position. This control allows the microjet to orient along the field lines using the magnetic torque exerted on its iron layers. The magnetic torque is calculated to be 2.6
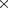
10^−13^ N.m, based on the characterized magnetic dipole moment of the microjet and the maximum magnetic fields (2 mT) used throughout the experimental work. This magnetic torque allows the microjet to overcome a drag torque of 3.3
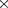
10^−19^ N.m, based on an angular velocity of 25 rad/sec. Our control system uses the magnetic torque only to keep the microjet within the vicinity of the reference position due to the self-propulsion force of the microjet that cannot be controlled during a motion control task. The unmodeled dynamics due to the wall and surface effects and microjet-bubbles interactions are mitigated using a sliding-mode control system. This control system achieves 14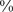 higher positioning accuracy than a magnetic-based PD control system. The accuracy provided by the proposed closed-control system could allow microjets to be used in micro-manipulation, micro-assembly, micro-actuation, and applications that are not yet conceived.

## References

[1] Paxton WF, Kistler KC, Olmeda CC, Sen A, St. Angelo SK, et al (2004) Catalytic nanomotors: autonomous movement of striped nanorods. Journal of the American Chemical Society 126: 13424–13431. 15479099 10.1021/ja047697z

[2] Fournier-Bidoz S, Arsenault AC, Manners I, Ozin GA (2004) Synthetic self-propelled nanorotors. Chemical Communication 441: 441–443. 10.1039/b414896g 15654363

[3] Howse JR, Jones RAL, Ryan AJ, Gough T, Vafabakhsh R, et al (2007) Self-Motile colloidal particles: from directed propulsion to random walk. Physical Review Letters 99: 048102. 17678409 10.1103/PhysRevLett.99.048102

[4] Mei YF, Huang G, Solovev AA, Urena EB, Monch I, et al (2008) Versatile approach for integrative and functionalized tubes by strain engineering of nanomembranes on polymers. Advanced Materials 20: 4085–4090.

[5] Solovev AA, Mei YF, Urena EB, Huang G, Schmidt OG (2009) Catalytic microtubular jet engines self-propelled by accumulated gas bubbles. Small 5: 1688–1692. 19373828 10.1002/smll.200900021

[6] Zhang L, Abbott JJ, Dong L, Kratochvil BE, Bell D, et al (2009) Artificial bacterial flagella: fabrication and magnetic control. Applied Physics Letters 94: 064107.

[7] Ghosh A, Fischer P (2009) Controlled propulsion of artificial magnetic nanostructured propellers. Nano Letters 9: 2243–2245. 19413293 10.1021/nl900186w

[8] Abbott JJ, Peyer KE, Lagomarsino MC, Zhang L, Dong L, et al (2009) How should microrobots swim? The International Journal of Robotics Research 28: 1434–1447.

[9] Nelson BJ, Kaliakatsos IK, Abbott JJ (2010) Microrobots for minimally invasive medicine. Annual Review of Biomedical Engineering 12: 55–85. 10.1146/annurev-bioeng-010510-103409 20415589

[10] Gibbs JG, Zhao Y-P (2009) Autonomously motile catalytic nanomotors by bubble propulsion. Applied Physics Letters 94: 163104.

[11] Solovev AA, Sanchez S, Pumera M, Mei YF, Schmidt OG (2010) Magnetic Control of Tubular Catalytic Microbots for the Transport, Assembly, and Delivery of Micro-objects. Advanced Functional Materials 20: 2430–2435.

[12] Mei YF, Solovev AA, Sanchez S, Schmidt OG (2011) Rolled-up nanotech on polymers: from basic perception to self-propelled catalytic microengines. Chemical Society Review 40: 2109–2119. 10.1039/c0cs00078g 21340080

[13] Sanchez S, Solovev AA, Schulze S, Schmidt OG (2010) Controlled manipulation of multiple cells using catalytic microbots. Chemical Communication 47: 698–700. 10.1039/c0cc04126b 21088790

[14] Keuning JD, de Vries J, Abelmann L, Misra S (2011) Image-based magnetic control of paramagnetic microparticles in water. Proceedings of the IEEE/RSJ International Conference of Robotics and Systems (IROS) 421–426.

[15] Floyd S, Pawashe C, Sitti M (2009) Two-dimensional contact and noncontact micromanipulation in liquid using an untethered mobile magnetic microrobot. IEEE Transactions on Robotics 25: 1332–1342.

[16] Kummer MP, Abbott JJ, Kartochvil BE, Borer R, Sengul A, et al (2010) OctoMag: an electromagnetic system for 5-DOF wireless micromanipulation. IEEE Transactions on Robotics 26: 1006–1017.

[17] Kratochvil BE, Kummer MP, Erni S, Borer R, Frutiger DR, et al (2010) MiniMag: a hemispherical electromagnetic system for 5-DOF wireless micromanipulation. Proceedings of the 12th International Symposium on Experimental Robotics-Springer Tracts in Advanced Robotics

[18] Pawashe C, Floyd S, Diller E, Sitti M (2012) Two-dimensional autonomous microparticle manipulation strategies for magnetic microrobots in fluidic environments. IEEE Transactions on Robotics 28: 467–477.

[19] Bell DJ, Leutenegger S, Hammar KM, Dong LX, Nelson BJ (2007) Flagella-like propulsion for microrobots using a nanocoil and a rotating electromagnetic field. Proceedings of the IEEE International Conference in Robotics and Automation (ICRA) 1128–1133.

[20] Sendoh M, Ajiro N, Ishiyama K, Inoue M, Arai KI, et al (1999) Effect of machine shape on swimming properties of the spiral-type magnetic micro-machine. IEEE Transactions on Magnetics 35: 3688–3690.

[21] Blakemore RP (1975) Magnetotactic bacteria. Science 190: 377–379. 170679 10.1126/science.170679

[22] Lu Z, Martel S (2007) Controlled bio-carriers based on magnetotactic bacteria. Proceedings of the IEEE International Conference on Solid-State Sensors, Actuators and Microsystems 683–686.

[23] Martel S, Mohammadi M (2010) Using a swarm of self-propelled natural microrobots in the form of flagellated bacteria to perform complex micro-assembly tasks. Proceedings of the IEEE International Conference on Robotics and Automation (ICRA) 500–505.

[24] Martel S, Tremblay CC, Ngakeng S, Langlois G (2006) Controlled manipulation and actuation of micro-objects with magnetotactic bacteria. Applied Physics Letters 89: 233904.

[25] Frankel RB, Williams TJ, Bazylinski DA (2007) Magneto-aerotaxis. Magnetoreception and Magnetosomes in Bacteria Microbiology Monographs 3: 1–24.

[26] Khalil ISM, Pichel MP, Abelmann L, Misra S (2013) Closed-loop control of magnetotactic bacteria. The International Journal of Robotics Research 32: 636–648.

[27] Wang J, Gao W (2012) Nano/Microscale motors: biomedical opportunities and challenges. ACS Nano 6: 5745–5751. 22770233 10.1021/nn3028997

[28] Wang J, Manesh KM (2010) Motion Control at the Nanoscale. Small 6: 338–345. 20013944 10.1002/smll.200901746

[29] Utkin VI, Chang H (2002) Sliding mode control on electro-mechanical systems. Mathematical problems in Engineering 8: 4–5.

[30] Bahaj AS, James PAB (1993) Characterisation of magnetotactic bacteria using image processing techniques. IEEE Transactions on Magnetics 29: 3358–3360.

[31] Bahaj AS, James PAB, Moeschler FD (1996) An alternative method for the estimation of the magnetic moment of non-spherical magnetotactic bacteria. IEEE Transactions on Magnetics 32: 5133–5135.

[32] Schmidt OG, Eberl K (2001) Thin solid films roll up into nanotubes. Nature 410: 168. 11242068 10.1038/35065525

[33] Chemla YR, Grossman HL, Lee TS, Clarke J, Adamkiewicz M, et al (1999) A new study of bacterial motion: superconducting quantum interference device microscopy of magnetotactic bacteria. Biophysical Journal 76: 3323–3330. 10354458 10.1016/S0006-3495(99)77485-0 PMC1300302

